# Supplementary material for: Evaluation of a Hybrid Moving Bed Biofilm Membrane Bioreactor and a Direct Contact Membrane Distillation System for Purification of Industrial Wastewater
Source: Membranes (Basel). 2022 Dec 22;13(1):16. doi: 10.3390/membranes13010016 (PMC9863120; doi:10.3390/membranes13010016)
Supplement: Supplementary file 1 [file membranes-13-00016-s001.zip › membranes-2016263-supplementary.pdf]

Table S1. Characteristics of the industrial wastewater streams at the Industrial City Wastewater Treatment Plant (Modon JICWTP, Jeddah)

Parameter	Secondary clarifier effluent	Secondary clarifier effluent	Primary treatment effluent	Instrument/method
Date of collection	14/08/2021	15/09/2021	23/11/2021	
**Physical Parameters**				
Appearance	Not clear	Not clear	Not clear	Visual inspection
Colour	Greenish yellow	Light yellow	Light black	Visual inspection
pH	9.44	8.9	4.52	pH meter
Conductivity (EC) (μS/cm)	1670	1666	1964	Conductivity meter
Salinity (ppt)	0.8	0.8	1.0	Conductivity meter
TDS (Total dissolved solids) (mg/L)	818	816	962	Conductivity meter
TSS (Total suspended solids) (mg/L)	89.6	103.5	524.1	Gravimetry
Turbidity (NTU)	1.6	1.3	40	Turbidity meter
TOC (Total organic carbon) (mg/L)	12	12.5	286.3	Gravimetry
**Anions & Cations**				
Fluoride (F) (ppm)	2.3	2	0.3	Ion Chromatography
Chloride (Cl) (ppm)	304.1	295.2	341.9	Ion Chromatography
Nitrite (NO2)	0	0	1.315 ppm	Ion Chromatography
Bromide (Br) (ppm)	1.1	1.0	4.6	Ion Chromatography
Nitrate (NO3) (ppm)	0	0	12.7	Ion Chromatography
Phosphate (PO4) (ppm)	0	0	0.3	Ion Chromatography
Sulphate (SO4) (ppm)	36.8	48.5	109.9	Ion Chromatography
Sodium (Na) (ppm)	286.2	279.7	313.5	Ion Chromatography
Potassium (K) (ppm)	24.3	25.1	31.9	Ion Chromatography
Magnesium (Mg) (ppm)	5.5	5.2	7.3	Ion Chromatography
Calcium (Ca) (ppm)	16.6	20.2	24.3	Ion Chromatography
Elements / Metals:				

Ag (silver) (ppm)	0.007	0.007	Not detected	ICP-OES
Al (aluminum) (ppm)	0.41	0.57	1.89	ICP-OES
As (arsenic) (ppm)	0.047	Not detected	Not detected	ICP-OES
Cr (chromium) (ppm)	0.005	0.002	Not detected	ICP-OES
Cu (copper) (ppm)	0.0758 ppm	0.0772 ppm	0.0927 ppm	ICP-OES
Fe (iron) (ppm)	0.022	0.020	0.303	ICP-OES
Mo (molybdenum) (ppm)	0.060	0.064	0.018	ICP-OES
Ni (nickel) (ppm)	0.009	Not detected	0.007	ICP-OES
Sb (antimony) (ppm)	0.029	0.029	Not detected	ICP-OES
Se (selenium) (ppm)	0.054	0.036	Not detected	ICP-OES
Sr (strontium) (ppm)	0.058	Not detected	0.127	ICP-OES
V (vanadium) (ppm)	0.007	0.007	Not detected	ICP-OES
Zn (zinc) (ppm)	0.084	0.064	0.16	ICP-OES
